# Art therapy and psychosis – experiences from the University psychiatric hospital „Sveti Ivan“

**DOI:** 10.1192/j.eurpsy.2022.824

**Published:** 2022-09-01

**Authors:** I. Barun, S. Vuk Pisk, D. Šago, T. Zadravec, I. Filipcic

**Affiliations:** 1 University psychiatric hospital “Sveti Ivan”, Department Of Integrative Psychiatry, Zagreb, Croatia; 2 University psychiatric hospital “Sveti Ivan”, Day Hospital For Psychotic Disorders, Zagreb, Croatia; 3 University psychiatric hospital “Sveti Ivan”, Day Hospital For Integrative Psychiatry, Zagreb, Croatia

**Keywords:** art therapy, clinical practice, Psychosis

## Abstract

**Introduction:**

The language of visual arts speaks to us in a way that words cannot. Acknowledging the therapeutic effects of artistic expression, art therapy – a psychotherapeutic approach that integrates expressive characteristics of art and explorative characteristics of psychotherapy – has developed. From its beginnings, it has been used with people with psychotic disorders and is enlisted in NICE guidelines as psychological therapy for psychosis and schizophrenia.

**Objectives:**

To understand and to activate the potential of artistic expression in people with psychotic disorders treated on acute ward, in day hospitals and as a form of long-term therapy in the Patients club of the University psychiatric hospital „Sveti Ivan“.

**Methods:**

Art therapy programme is conducted separately on acute ward (Ward for integrative psychiatry), day hospitals (Day hospital for integrative psychiatry and Day hospital for psychotic disorders) and in the Patients club with patients with psychotic disorders. The workshops are adjusted for people with psychotic disorders to enable them to strengthen their sense of self, to empower them and to express their authentic feelings in a safe environment.

**Results:**

The artwork of people who have taken part in the art therapy programmes for psychosis of the University psychiatric hospital „Sveti Ivan“ will be presented and will serve as an example of an art therapy process, therapeutic goals, as well as the significance of this method for psychotic disorders.

**Conclusions:**

Art therapy can be of great benefit for people with psychosis both on acute wards and as a long-term therapy.

**Disclosure:**

No significant relationships.

